# Pregnancy outcomes following non-screening of tuberculosis at primary healthcare facilities

**DOI:** 10.4102/hsag.v29i0.2537

**Published:** 2024-05-20

**Authors:** Violet M. Chewe

**Affiliations:** 1 Department of Advanced Nursing Science, Faculty of Health Sciences, University of Venda, Thohoyandou, South Africa

**Keywords:** midwives, pregnancy outcomes, primary healthcare, non-screening, tuberculosis

## Abstract

**Background:**

In South Africa, tuberculosis (TB) screening should be offered to all pregnant women at each antenatal care (ANC) visit. Therefore, access to TB screening for women and their families is crucial through maternity and child health services.

**Aim:**

The study aimed to explore the repercussions of the non-screening of TB among pregnant women in the Capricorn District of Limpopo province.

**Setting:**

The study was conducted in the selected primary healthcare facilities in the Capricorn District, Limpopo province, at selected primary healthcare facilities. Midwives employed within the primary healthcare facilities of Limpopo, offering ANC to pregnant women comprised the population.

**Methods:**

The study was carried out using a qualitative exploratory research methodology. Ten participants were selected using purposive sampling method. As a result of limitations on coronavirus disease, data were gathered through in-depth, semi-structured virtual interviews with individual participants. Data analysis was employed manually using Tesch’s method.

**Results:**

Two themes emerged from the data analysis: obstetric outcomes because of non-screening of TB and suggestions to enhance TB screening during ANC.

**Conclusion:**

The study’s findings revealed that non-screening of TB among pregnant women may lead to severe pregnancy outcomes for both the mother and the unborn child should the woman be suffering from TB.

**Contribution:**

The Department of Health may employ the results of this study to develop strategies that might be implemented to enhance TB screening among pregnant women and improve pregnancy outcomes for women diagnosed with TB.

## Introduction and background

Tuberculosis (TB) screening entails detecting active TB disease using examinations, testing, or other quick procedures, World Health Organization (WHO 2023:13). In 2021, 10.6 million cases of TB were reported globally (WHO 2023:1). Furthermore, according to the WHO (2023:3), 1.6 million people globally passed away from TB in 2021. Women are diagnosed with one-third of the 10 million new cases of TB each year (Mathad et al. 2022:3). However, epidemiologic data on TB conditions during pregnancy is not routinely collected. Without carefully gathering data, global modelling studies project 200 000 incidence TB cases during pregnancy each year (Sugarman et al. 2014:710).

Some countries have tried several strategies to improve TB screening among pregnant women; hence, TB screening was given less attention (WHO 2022:2). For instance, SA embraced the Sustainable Development Goals (SDGs) of WHO (2018:11) to reduce the spread of TB among the public by 2030. The 90-90-90 plan, also known as the ‘End TB’ strategy, was launched in SA in December 2014 to meet the Joint United Nations Program on HIV/AIDS (UNAIDS) ‘fast track’ targets (Malaza et al. 2016:10). This entails screening 90% of individuals in the key demographics, initiating TB treatment for 90% of those who receive a TB diagnosis and ensuring that 90% of patients who begin treatment complete it. Early TB disease detection and treatment can reduce morbidity and mortality among pregnant women and newborns.

All pregnant women should undergo TB screening at each antenatal appointment, according to the Guidelines on Maternity Care in SA (2016), the Confidential Report on Maternal Mortality in SA (2018), and the Guideline for the Prevention of Mother-to-Child Transmission of Communicable Infections (2018). Massyn et al. (2020:9) estimated that in 2019/2020, 90.7% of pregnant women in Limpopo registered for antenatal care (ANC) at less than 20 weeks of gestation. This suggests that women schedule ANC earlier in their pregnancies so that TB screening can be performed and a diagnosis is made early.

During routine visits to primary healthcare (PHC) facilities, the researcher noticed that nearly all the pregnant women’s antenatal cards lacked evidence of TB screening. As an advanced midwife and a basic ANC facilitator, the researcher frequently attended meetings on maternal mortality in the province’s two provincial hospitals. In those meetings, non-pregnancy-related infections, of which TB is most common, account for most maternal deaths discussed. Further findings are that TB screening does not usually reflect when reviewing ANC cards during the meetings. The understanding is that TB screening was omitted, and pregnant women were only diagnosed with the disease after it was already advanced.

Multiple studies have been conducted on TB infections; for instance, Odayar et al. (2018:762) emphasized enhancing TB prevention in pregnant women. In contrast, Fowks et al. (2016:6) focused on identifying common structural obstacles to providing integrated ANC, which includes TB screening. Of the literature consulted, midwives’ reflections on TB in pregnant women received less attention.

Limpopo is one of the provinces in SA that is burdened by maternal mortality related to non-pregnancy related infections, with TB infection being the leading cause (SA 2017:12). According to SA (2017:26), pregnant women are dying from TB infection, which is preventable and treatable, despite the established guidelines for TB screening of pregnant women (SA 2023:21). The researcher concluded that further research on repercussions on the pregnancy outcomes of non-screening of TB in pregnant women was crucial as a way of understanding the reason behind high maternal mortalities and suggesting corrective measures.

The study aimed to explore the repercussions of the non-screening of TB among pregnant women in Limpopo province.

## Research methods and design

### Research design

The study used a qualitative explorative-descriptive design. The researcher comprehended repercussions regarding the non-screening for TB during ANC in the province of Limpopo. Although there are guidelines in place to manage TB, the researcher was interested in finding out why the local area had high rates of maternal death related to TB infection. The study’s design was acceptable as it described the repercussions of the omission of screening for TB in pregnant women.

### Study setting

The study occurred in the selected local area under the Capricorn District of Limpopo province. The selected local area consists of seven PHC clinics. purposive sampling was used to select participants for this study Capricorn District of Limpopo province, the local area, and the seven PHC clinics. The study was conducted at all seven PHC clinics because of a limited number of midwives and high maternal mortality related to TB infection (SA 2018:30).

### Population

The population for this study consisted of all midwives currently employed in public PHC clinics in Capricorn District of Limpopo province. The total number of midwives employed at the time of data collection in the selected public primary health facilities was 42. The operational managers of the institutions provided support in determining the population. Table 1 provides a summary of the population.

**TABLE 1:** Population for the selected local area.

Clinic name	Total number of midwives
Clinic A	05
Clinic B	06
Clinic C	09
Clinic D	05
Clinic E	06
Clinic F	06
Clinic G	05

**Total**	**42**

*Source:* Chewe, V.M., 2021, ‘Tuberculosis screening among human immune deficiency virus positive pregnant women in Limpopo Province’, MA Dissertation, University of South Africa, Pretoria

### Sampling and sample size

Purposive sampling was used to select participants for this study. Participants included midwives with experience dealing with pregnant women employed at the PHC clinics in the chosen locality. Ten participants were interviewed for this study and data saturation determined the sample size.

### Data collection

The method for gathering data in this study was one-on-one, in-depth individual virtual video interviews such as zoom and Teams. The researcher collected data between December 2020 and July 2021. The participants selected a day and time that suited them. As a result, the researcher could notice nonverbal cues and body language. The limitations presented by the coronavirus disease 2019 (COVID-19) during data collection led to the use of this approach. Bias was avoided by using semi-structured questions for all participants. During the interview, probing was guided by the participant’s answers, not the researcher’s interests. Probing also assisted the researcher, in obtaining rich, valuable data.

### Data analysis and management

Tesch’s eight steps for data analysis were used to identify and develop themes and subthemes. The researcher used data organisation to categorize, concentrate, delete, and arrange data to draw and verify findings. The researcher protected and preserved all data records in their original configuration. The transcripts, field notes, and audiotapes were safely preserved to ease the scientific pressure of data processing and minimize contamination. After the coder independently coded the data, the researcher and coder agreed on the themes and subthemes.

### Measures to ensure trustworthiness

To assure trustworthiness, the researcher listened to collected data, which was verbatim transcribed, and noticed perceptions as they occurred. As a result, the researcher reviewed the participant’s transcripts several times until she understood them.

### Ethical considerations

University South Africa Research Committee (HSHDC/934/2019) and the Limpopo Department of Health (LP-202001-015) provided ethical clearance for the study to proceed. The Capricorn District and clinic managers also gave their approval. The researchers obtained the participants’ phone numbers from their operational managers, who facilitated the recruitment process. The researcher personally called each participant to recruit them. Furthermore, participants were given information and a pamphlet regarding the study and its objectives via email before the study. All participants who agreed to participate in the study provided written informed consent before participation.

Ethical principles, such as autonomy, were adhered to, which gave participants the freedom to decide whether to engage in the study without facing penalties. The principle of beneficence and maleficence were observed in the study by protecting participants from harm or discomfort during data collection. In addition, the participants were told they could discontinue the research without facing consequences if they felt so. And lastly, the principle of justice was also maintained. The research attributes were the only selection criteria used to select study participants, and each eligible person had an equal chance of participation. Participants were given the P1 to P10 pseudonyms to protect their privacy and anonymity.

## Results

### Demographic of participants

Ten participants were interviewed: nine midwives and one accoucheur aged between 30 years – 47 years. The participants had 6 years – 17 years of experience working with pregnant women. Table 2 summarises the demographic information of participants.

**TABLE 2:** Participants’ demographic information.

Participant	Age (years)	Gender	Category	Academic qualifications	Years of experience
1	37	Female	Midwife	Diploma in Nursing Science and Midwifery	10
2	35	Female	Midwife	Diploma in Nursing Science and Midwifery	06
3	43	Male	Accoucheur	Diploma in Nursing Science and Midwifery	12
4	45	Female	Midwife	Diploma in Nursing Science and Midwifery	09
5	35	Female	Midwife	Diploma in Nursing Science and Midwifery	10
6	33	Female	Midwife	Diploma in Nursing Science and Midwifery	07
7	34	Female	Midwife	Diploma in Nursing Science and Midwifery	07
8	48	Female	Midwife	Bachelor’s degree in nursing science and Midwifery	17
9	40	Female	Midwife	Diploma in Nursing Science and Midwifery	11
10	30	Female	Midwife	Diploma in Nursing Science and Midwifery	06

*Source:* Chewe, V.M., 2021, ‘Tuberculosis screening among human immune deficiency virus positive pregnant women in Limpopo Province’, MA Dissertation, University of South Africa, Pretoria

## Themes and sub-themes

The two themes, obstetric outcomes, and suggestions to enhance TB testing during prenatal care, and four subthemes emerged from data collection. The themes and sub-themes are shown in Table 3.

**TABLE 3:** Themes and sub-themes.

Themes	Sub-themes
1. Obstetric outcomes	1.1. The upsurge in neonatal complications and perinatal mortality 1.2. Increased maternal complications and maternal mortality
2. Suggestions to enhance TB testing during antenatal care (ANC)	2.1. Proper delegation of responsibilities 2.2. Proper sorting of patients

*Source*: Chewe, V.M., 2021, ‘Tuberculosis screening among human immune deficiency virus positive pregnant women in Limpopo Province’, MA Dissertation, University of South Africa, Pretoria

TB, tuberculosis.

### Theme 1: Obstetric outcomes

Participants were alarmed by the potential consequences of an untreated pregnant TB woman giving delivery. They indicated a rise in maternal and newborn complications.

#### Sub-Theme 1.1: The upsurge in neonatal complications and perinatal mortality

Participants expressed their sorrow about the likelihood that a newborn whose mother has a TB infection could be born with a range of problems, including congenital TB infection, specific deformities, and neonatal death. Participant reports state:

‘Is … the unborn baby is not safe. The unborn baby is not safe … [*Moment of silence*] … because uhm … this can lead to the death of the fetus in utero. Yes, this may result in a uterine death … and this may also result in the baby not growing well in the utero.’ (P1)

‘Because the mother is not gaining weight or is experiencing health issues, which will cause us to have low birth weight babies since the mother has TB; however, if we obtain … The health of the pregnant ladies will return to normal if we can help the mother by diagnosing her TB early and initiating treatment.’ (P10)

#### Sub-theme 1.2 Increased maternal complications and maternal mortality

Participants in this study cautioned that if pregnant women are not screened for TB, other maternal issues, such as mortality and severe illness, may develop in pregnant women in case they are infected. The participant said the following:

‘So we might lose the patient, the patient might die, because we have detected the … that infection late. Yes, we will be having uhm … we are going to have maternal death in the facility due to the omission of TB screening.’ (P7)

‘Ohk. For the few that I do know, this is pneumonia uhm … [*silence*] I believe. Oh, and I almost forgot, but even the … It can be due to what they say … TB that is MDR [*multi-drug resistant*]. I don’t know how to go into further detail, but the result is death.’ (P6)

### Theme 2: Suggestions to encourage tuberculosis testing during antenatal care

Participants offered ideas for enhancing the effectiveness of TB screening among pregnant women in the facilities.

#### Sub-theme 2.1: Proper delegation of responsibilities

The participants proposed that delegation be strengthened and effectively used to ensure that a person in charge of daily TB screening is delegated. The patient hinted at the following:

‘I think a delegation … [*pause*] can improve it. Somebody who concentrates on it the whole day … and just brings patients. I think that would help … because … except for that, well, I can say ohk [*okay*] today I’m gonna [*will*] screen, but I’ll only screen maybe five, from there, I’m no longer screening them.’ (P5)

‘Well … [*laughter*]. There’s a matter of assigning a certain person who will deal with such matters, but if that person recognizes, “Oh, this is my task for the day,” and she admits it as such and signs for it, it will be simple.’ (P9)

#### Sub-theme 2.2: Proper sorting of patients

Triaging is used in most healthcare settings to prioritise and group patients according to the priority type of treatment they require. Doing triaging would make the midwives less likely to miss pregnant women for TB screening. The participants’ response is as follows:

‘I think the most important thing is the triage … [*Silence*] I think the most important thing is the triage. When they do, the triage we should triage them so that they should undergo the screening, and the screening should be each visit to the ANC one. We must make sure that they are screened.’ (P2)

‘Yes. With triaging, um … [*frown*] When patients arrived at the clinic, we should triage them to make sure that, among other things, those who needed immediate attention were given priority. In the facility, pregnant women are given priority.’ (P4)

A total of two themes and four sub-themes were identified from the study results, as illustrated in Table 1. The participant responses additionally confirmed the results.

## Discussion

Most maternal deaths in the province of Limpopo are caused by non-pregnancy-related infections, of which TB is the main cause (SA 2019:23). Pregnancy-related TB screening may help with early detection and treatment. This could help lower the rate of maternal death in Limpopo province caused by TB infections. The findings of the study generated several repercussions on the effects of not screening pregnant women for TB. The responses covered a wide range of areas, including non-screening outcomes for TB during pregnancy and the proposed suggestions to enhance TB screening during ANC.

The participants emphasised some of the consequences of not screening TB during pregnancy for both the mother and the unborn child. Given that TB is fatal, particularly in neonates, this could result in perinatal morbidity and mortality. Participants further raised worries about the possibilities of congenital TB, intrauterine development limits, underweight newborns, and premature babies. This is in line with studies by Alene, Jegnie and Adanec (2021:8), Phoswa, Eche and Khaliq (2020:104), which found that pregnant women with TB infection are more likely to have poor outcomes for the foetus. According to Bada et al. (2020:10), a delayed diagnosis of TB may result in additional treatment and specialized care, which could get expensive for the state.

Concerning the results of not screening for TB during ANC, the study further demonstrated that delaying TB screening and receiving a diagnosis later in pregnancy can have detrimental effects on the pregnant woman. Multi-drug resistant TB (MDR-TB), inadequate weight gain, poor prognosis, and maternal death are some concerns indicated. This study’s results are consistent with those of other 2020 and 2021 investigations that discovered a link between TB infection during pregnancy and severe maternal complications (Bares & Swinddells 2020:4; Hamda et al. 2020:76; Orazuluke et al. 2021:169). Given that pregnant women are particularly vulnerable, early diagnosis and rapid care can be initiated with TB screening, preventing complications, including maternal mortality.

The study provided suggestions, for enhancing TB screening during ANC. Participants suggested implementing regular staff delegation for TB screening in pregnant women. The participants anticipate that the delegation technique would help to divide the workload and keep pregnant women from being overlooked regarding TB screening. Employees who accept responsibility for their assigned responsibilities will be able to better the quality of TB screening services for pregnant women. According to Shousha, Gad and Shokier (2023:194), delegation improves organisational effectiveness by helping nurse managers set priorities and manage their time. Crevacore et al. (2023:894) also state that registered midwives should ensure the staff assigned with tasks are adequately trained.

These days, most individuals seeking ANC services know their rights and show up at the health facilities prepared. According to the study by Kloester et al. (2019:10), midwives encounter challenges when patients report to the appropriate authorities about problems. The drawing of a daily delegation with an emphasis on TB screening is necessary to ensure that every pregnant woman who enters the facility is screened to avoid unnecessary lawsuits.

The study’s findings further suggested sorting patients as they arrive at the clinic to ensure that pregnant women receive their TB screenings. To avoid missing other patients, midwives should better manage their patient load by grouping patients according to their needs and priorities. Participants believed arranging patients at the facilities based on their designated classifications would enhance TB screening for pregnant women as everyone would be assisted based on their allocated classifications. Stott and Moosa (2019:6) emphasise the importance of developing a workable triage procedure. According to Zaboli et al. (2022:7), daily triaging is further anticipated to reduce errors and improve performance in healthcare institutions. Therefore, health facilities are advised to use a triage method to arrange patients based on their needs to make TB screening easier for pregnant women.

The study discussed the obstetric outcomes of non-screening for TB and the recommendations for enhancing antenatal TB screening. With TB screening at each antenatal visit, prevention can be carried out through the initiation of TB prophylaxis in HIV-positive pregnant women, early diagnosis, as well as prompt management for TB-infected pregnant women. This can be performed to prevent severe complications for both the mother and the unborn child, including perinatal and maternal mortalities.

### Limitations

- The research was limited to one local area of the South African province of Limpopo.
- Data collection was performed using a virtual video call due to COVID-19 restrictions.

### Recommendations

#### Nursing practice

- Operational managers should strengthen delegation methods and proper sorting of patients to prioritise screening pregnant women for TB.
- Midwives should prioritise TB screening in the antenatal period for early diagnosis and initiation of treatment to prevent maternal and neonatal complications.

#### Nursing research

- The study may be expanded to other locations, districts, and SA provinces to understand this phenomenon better.

#### Nursing education

- Guidelines should be developed and incorporated into the nursing curricula to support midwives implementing TB screening among pregnant women.

## Conclusion

The study’s findings revealed that non-screening of TB among pregnant women may lead to severe pregnancy outcomes for both the mother and the unborn child should the woman be suffering from TB. Participants emphasised increased severe newborn and maternal complications because of non-screening of TB during pregnancy. The results also offered several improvements that may be implemented for antenatal TB screening, such as delegation and triaging. As a result, midwives in PHC ought to screen pregnant women for TB at every visit to ensure early identification and timely treatment to promote positive obstetric outcomes and prevent newborn and maternal mortality.
